# Low-Concentration Ciprofloxacin Selects Plasmid-Mediated Quinolone Resistance Encoding Genes and Affects Bacterial Taxa in Soil Containing Manure

**DOI:** 10.3389/fmicb.2016.01730

**Published:** 2016-11-01

**Authors:** Ting Huang, Ying Xu, Jie Zeng, Dong-Hao Zhao, Liang Li, Xiao-Ping Liao, Ya-Hong Liu, Jian Sun

**Affiliations:** ^1^National Risk Assessment Laboratory for Antimicrobial Resistance of Animal Original Bacteria, College of Veterinary Medicine, South China Agricultural UniversityGuangzhou, China; ^2^Guangdong Provincial Key Laboratory of Veterinary Pharmaceutics Development and Safety Evaluation, South China Agricultural UniversityGuangzhou, China; ^3^Jiangsu Co-innovation Center for Prevention and Control of Important Animal Infectious Diseases and ZoonosesYangzhou, China

**Keywords:** ciprofloxacin, manure, soil, PMQR-encoding genes, bacterial taxa

## Abstract

The spread of antimicrobial resistance in environment is promoted at least in part by the inappropriate use of antibiotics in animals and humans. The present study was designed to investigate the impact of different concentrations of ciprofloxacin in soil containing manure on the development of plasmid-mediated quinolone resistance (PMQR) – encoding genes and the abundance of soil bacterial communities. For these studies, high-throughput next-generation sequencing of 16S rRNA, real-time polymerase chain reaction and standard microbiologic culture methods were utilized. We demonstrated that the dissipate rate of relative abundances of some of PMQR-encoding genes, such as *qnrS*, *oqxA* and *aac(6^′^)-Ib-cr*, were significantly lower with ciprofloxacin 0.04 and 0.4 mg/kg exposure as compared to no-ciprofloxacin control and ciprofloxacin 4 mg/kg exposure during 2 month. Also, the number of ciprofloxacin resistant bacteria was significantly greater in ciprofloxacin 0.04 and 0.4 mg/kg exposure as compared with no-ciprofloxacin control and the ciprofloxacin 4 mg/kg exposure. In addition, lower ciprofloxacin concentration provided a selective advantage for the populations of *Xanthomonadales* and *Bacillales* in orders while *Agrobacterium*, *Bacillus*, *Enterococcus*, and *Burkholderia* in genera. These findings suggest that lower concentration of ciprofloxacin resulted in a slower rate of PMQR-encoding genes dissipation and selected development of ciprofloxacin-resistant bacteria in soil amended with manure.

## Introduction

The majority of antibiotics are poorly absorbed in animal gut, and significant amount of antibiotics is excreted into the environment through feces and urine (Chee-Sanford et al., 2009). In China, manures are commonly applied to agricultural land to recycle their plant nutrients. The agricultural land has accumulated a statistically significant higher antibiotics concentration than conventional open croplands because the use of animal manure (Zhang et al., 2016). In addition, it is well-known that different residual concentrations of ciprofloxacin have been found in manure-amended environment (Zhao et al., 2010; Leal et al., 2012; Li et al., 2014). For instance, ciprofloxacin had a higher detection frequency and maximum concentration in 20–60 cm soil layer than that in 0–20 cm soil layer (Wei et al., 2016). The potential of subinhibitory concentration of antibiotics to promote the development of antibiotic-resistance genes (ARGs) in complex bacterial communities (Berendonk et al., 2015). For example, even at levels that are considered safe according to the currently accepted Environmental Quality Standard, antibiotics can still select for ARGs (Negri et al., 2000; Hughes and Andersson, 2012; Sandegren, 2014).

Ciprofloxacin is the most widely used fluoroquinolone antibiotic (Pico and Andreu, 2007) which is active against a broad spectrum of Gram-negative and Gram-positive bacteria (Davis et al., 1996), and it has been detected in various composts and manures (Zhang et al., 2015). Some horizontally transferable elements might help account for the strong association between resistance to quinolones, such as plasmid-mediated quinolone resistance (PMQR) – encoding genes (Strahilevitz et al., 2009). PMQR – encoding genes have been found in the environment due to the selective pressure posed by fluoroquinolones (Chen et al., 2012). So far, PMQR has managed to achieve global distribution in a variety of plasmid environments and bacteria genera (Rodriguez-Martinez et al., 2011; Poirel et al., 2012; Wong et al., 2014; Guillard et al., 2015). Presently three types of PMQR-encoding genes have been identified and these correspond to the Qnr proteins encoded by *qnrA*, *qnrB*, *qnrC*, *qnrD* and *qnrS*, an aminoglycoside acetyltransferase variant capable of reducing ciprofloxacin activity encoded by *aac(6^′^)-Ib-cr* (Robicsek et al., 2006; Jones-Dias et al., 2016), and drug eﬄux pumps encoded by *qepA* and *oqxAB* (Hernandez et al., 2011). These mechanisms provide the low-level quinolone resistance to facilitate the emergence of higher-level resistance in the presence of quinolones (Rodriguez-Martinez et al., 2011). In studies of soil bacterial isolates, over 76% of the fluoroquinolones (levofloxacin and ciprofloxacin) resistance was shown to be mediated by eﬄux (Walsh and Duffy, 2013). Although, more studies focused on the effect of different classes of antibiotics as selective pressure on ARGs and resistant bacteria in soil containing manure (Heuer et al., 2011; Ding et al., 2014; Tang et al., 2015), it is also important to explore the relationship between different antibiotic concentrations in the environment and bacterial taxa and PMQR-encoring genes.

The aim of the present study was to investigate the fate of PMQR-encoding genes and the abundance of soil bacterial communities posed by different concentrations of ciprofloxacin in manure applied to soil. To our knowledge, this is the first report to investigate the effect of different antibiotic residue levels on the dissipation rates of PMQR-encoding genes and bacterial taxa after manure application.

## Materials and Methods

### Treatment

Manure samples were collected from healthy mature ciprofloxacin-free swine was introduced into arable soils. The soils were collected from arable land which had no known exposure to antibiotics and had minimal human-induced selective pressure. Manure (60 g) was incorporated into 1500 g of 2 mm sieved soil (4%, w/w) with or without antibiotics and served as treatment and control groups, respectively (Heuer et al., 2011). Three treatment groups each containing three replicates were set up as follows: 4 mg kg^-1^ ciprofloxacin (A), 0.4 mg kg^-1^ ciprofloxacin (B) and 0.04 mg kg^-1^ ciprofloxacin (C). The ciprofloxacin doses were selected for the current studies based on previous reports indicating the average concentration of ciprofloxacin was about 0.04–0.4 mg/kg in the soil environment (Li et al., 2014; Xu et al., 2015). In addition, ciprofloxacin at 4 mg/kg is a standard dose for treatment in pigs (Nguyen et al., 2014). A control group lacking ciprofloxacin was designated group D. Pots containing the above mixtures were incubated at 20°C for 60 days in the dark. Water was added to the soil surface twice a week in order to maintain the soil moisture content at approximately 55% of the soil’s water holding capacity. Soil samples were collected at days 0, 30, and 60 (Xiong et al., 2015b).

### UPLC–MS/MS Determination

Soil samples (1 g) from different groups and time points as described above were freeze-dried at -80°C until usage. For sample processing, these were spiked with 5 ml extraction buffer containing equal proportions of acetonitrile/phosphate at pH 3.0. Solid phase extraction was conducted according to a previously published method (Luo et al., 2010). Ciprofloxacin concentrations were determined by ultra-performance liquid chromatography–electrospray tandem mass spectrometry (UPLC–MS/MS). Concentrated extracts were separated on an Agilent 1200 liquid chromatograph (Santa Clara, CA, USA) using a Waters Quattro Micro triple quadrupole mass spectrometer in multiple reactions monitoring mode with electrospray ionization in positive-ion mode (Milford, MA, USA).

### DNA Extractions

DNA from soil samples was extracted using the Power Soil Kit (Mo Bio Laboratories, Inc., Carlsbad, CA, USA) according to the manufacturer’s instructions. Total DNA was quantified using a NanoDrop^®^ ND-2000 UV spectrophotometer (NanoDrop Technologies, Wilmington, DE, USA). Only DNA samples with A260/A280 > 1.7 and A260/A230 > 1.8 were used for further analysis. The extracts were stored at -20°C until use.

### Detection and Relative Quantification of ARGs

Polymerase chain reaction (PCR) was used to screen the major PMQR genes *qnrA*, *qnrB*, *qnrC*, *qnrD*, *qnrS*, *qepA*, *aac(6^′^)-Ib-cr*, and *oqxA* as previously described (Sun et al., 2014). To ensure reproducibility, three replicate assays for each sample were performed in parallel with positive and negative controls in each run. PMQR and 16S rRNA genes were further quantified by real time qPCR using SYBR Premix Ex Taq (TAKARA Bio, Otsushi, Japan) in a Bio-Rad iQ5 thermal cycler according to the manufacturer’s instructions (Hercules, CA, USA). A eubacterial 16S rRNA gene (Sun et al., 2014) was also quantified so that ARGs could be normalized to the total bacterial community. Cycling conditions were as follows: 94°C for 5 min followed by 35 cycles at 94°C for 1 min, 60°C for 1 min and 72°C for 1 min with a final extension at 72°C for 5 min. Melting curve analysis was performed for every assay from 60 to 95°C with 1°C intervals. Given the temporal variations caused by total bacterial community and overall extraction efficiencies, the copies of ARGs were normalized to the 16S rRNA gene copies (ARGs copies/16S rRNA gene copies, defined as relative abundance). All experiments were performed in triplicate and the standard error of the measurements was determined from these parallel data.

### Total Bacteria Counts and Enumeration of Ciprofloxacin-Resistant (CIPr) Bacteria

Colony-forming unit (CFU) counts of culturable bacteria recovered from soil were detected on Luria-Bertani (LB) agar. Soil samples (wet weight 5 g) were recovered in 45 ml of 0.9% NaCl by shaking at 120 rpm at 25°C for 1 h. Soil particles were then allowed to settle for approximately 15 min at room temperature. About 100 μL of serial 10-fold dilutions were plated on LB agar and incubated at 37°C for 3 days in triplicate. Plates containing 4 mg/L of ciprofloxacin were used to counted CIPr bacteria based on previous studies (Sun et al., 2014). The fraction of CIPr bacteria was calculated as the ratio of bacteria growing on plates supplemented with CIP to the number of bacteria growing on plates without CIP (Xu et al., 2015).

### Amplicon Sequencing

The V3+V4 (Abrahamsson et al., 2014; Jakobsson et al., 2014) hypervariable regions of 16S rDNA was PCR amplified from microbial DNA harvested from three replications for four different groups at day 60. The PCR products from the three replications were pooled together in equimolar ratios for amplicon sequencing. The gene-specific sequences used the 16S V3 and V4 region, 341F 5^′^-CCTACGGGNGGCWGCAG-3^′^ and 805R 5^′^-GACTACHVGGGTATCTAATCC-3^′^ (Abrahamsson et al., 2014; Jakobsson et al., 2014). The PCR conditions were as follows: one pre-denaturation cycle at 94°C for 4 min, 25 cycles of denaturation at 94°C for 30 s, annealing at 55°C for 45 s, and elongation at 72°C for 30 s, and one post-elongation cycle at 72°C for 5 min. The PCR amplicons were separated on 0.8% agarose gels and then extracted. Only PCR products without primer dimers and contaminant bands were used for sequencing. Amplicons were purified using AMPure X using the manufacturer’s instructions (Beckman Coulter, Inc., Mississauga, ON, Canada). Bar-coded V3 and V4 amplicons were sequenced using the 2 × 300 paired-end method by Illumina MiSeq with a seven-cycle index read. Sequences processing was performed using QIIME (version 1.6.0) to get clean data. Sequences with an average Phred (Q) score lower than 30, with ambiguous bases or homopolymer runs exceeding 6 bp, primer mismatches or sequence lengths shorter than 100 bp were removed. The consensus sequence was generated by FLASH (Fast length Adjustment of Short reads, v1.2.11) as following: only sequences with an overlap longer than 10 bp and without any mismatch were assembled according to their overlap sequence. Reads that could not be assembled were discarded. Barcode and sequencing primers were trimmed from the assembled sequence. The high quality paired-end reads were combined to tags based on overlap. The tags were clustered to operational taxonomic unit (OTU) by software USEARCH (v7.0.1090). OTU representative sequences were taxonomically classified using Ribosomal Database Project (RDP) Classifier v2.2 trained on the Greengenes database.

### Statistical Analyses

Copies of PMQR-encoding genes were normalized (PMQR-encoding gene copies/16S rRNA gene copies) and log transformed for linear-regression analysis using SPSS 19.0. Significant differences of relative ARG abundance and resistant bacteria among different groups were analyzed by ANOVA and Bonferroni correction using SPSS 19.0. Differential abundance of bacterial taxa between different groups were compared using Fisher’s exact test (Xiong et al., 2015a) at a statistical difference level of *p* < 0.05 and Bonferroni correction.

## Results

### Concentration of Ciprofloxacin

The residual concentration of ciprofloxacin in soil from all study groups at days 0, 30, and 60 are shown in **Table 1**. A significant time and concentration-dependent ciprofloxacin residues were observed in all three ciprofloxacin treated groups, but as expected, not in the control group.

**Table 1:** Ciprofloxacin concentrations at days 0, 30, and 60.

Treatment group	Concentrations (μg kg^-1^)		
	0 days	30 days	60 days
A	1331.34 ± 118.68	759.48 ± 21.61	502.66 ± 50.44
B	151.35 ± 16.50	103.92 ± 0.8	63.80 ± 1.56
C	21.48 ± 1.93	15.32 ± 0.66	9.82 ± 0.46
D	ND	ND	ND

*ND, not detected.*

### Abundance of PMQR-Encoding Genes

**FIGURE 1:**
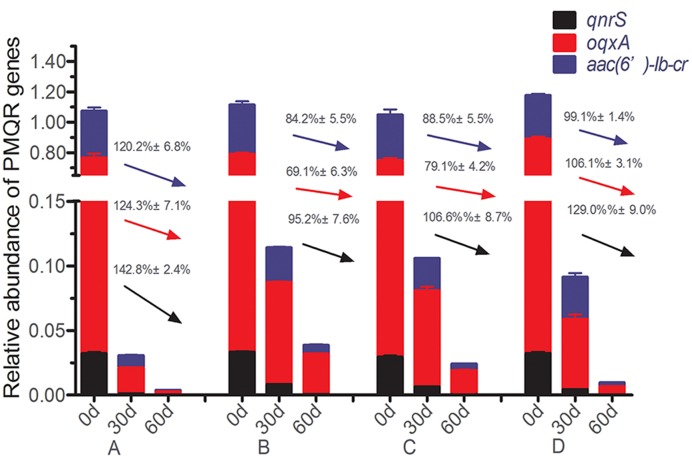
**Variations in the relative abundance of PMQR-encoding genes at days 0, 30, and 60 following treatments.** Dissipation rates are expressed by the values on the arrows [black for *qnrS*, red for *oqxA* and blue for *aac(6^′^)-Ib-cr*].

The relative abundances of three detectable PMQR genes [*qnrS*, *oqxA*, and *aac(6^′^)-lb-cr*] are showed in Supplementary Table S1. The current results showed that *qnrA*, *qnrB*, *qnrC*, *qnrD*, *qepA* were absent from all soil samples. At the day 60, the relative abundance of all detected PMQR-encoding genes in the treatment and control groups had significantly decreased as compared to day 0 (*p* < 0.05). For instance, the relative abundance of *qnrS* gene was decreased 129% in the control group from day 0 to day 60 (**Figure 1**). On the other hand, ciprofloxacin at 4 mg/kg had significantly higher dissipated rate of relative abundance of the three PMQR genes as compared to the control group. Therefore, ciprofloxacin at 4 mg/kg group had the lowest abundance at day 60 vs. all other tested groups (Supplementary Table S1). However, ciprofloxacin at 0.4 and 0.04 mg/kg concentrations, the dissipate rate of relative abundances of *qnrS*, *oqxA*, and *aac(6^′^)-lb-cr* were the lowest as compared to the control and ciprofloxacin 4 mg/kg groups (**Figure 1**).

### Shifts in the Number of Resistant Bacteria with Different Treatments

The proportion of bacterial communities resistant to ciprofloxacin in all study groups was similar (∼40%) at day 0, but decreased over 60 days (range from 42.66–46.06% to 14.67–29.93% for days 0 and 60, respectively) (**Supplementary Figure S1**). At days 30 and 60, ciprofloxacin-resistant bacteria communities in ciprofloxacin at 0.4 and 0.04 mg/kg groups were significantly higher than in ciprofloxacin at 4 mg/kg group, as well as the control group (*p* < 0.05).

### Bacteria Community Composition

**FIGURE 2:**
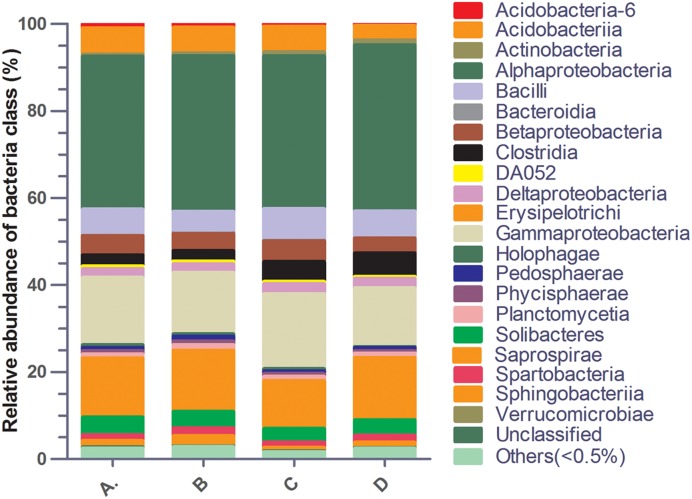
**Relative abundance of bacterial classes between different treatment groups. (A)** 4 mg kg^-1^ ciprofloxacin; **(B)** 0.4 mg kg^-1^ ciprofloxacin; **(C)** 0.04 mg kg^-1^ ciprofloxacin; **(D)** 0 mg kg^-1^, as control.

A total of 155,697 reads of the V3–V4 regions of bacterial 16S rDNA genes were obtained from a total of four soil samples. The dominant classes were *Alphaproteobacteria* (35.08–38.11%), *Gammaproteobacteria* (13.49–17.21%) and *Saprospirae* (11.01–14.28%) in all soil samples (**Figure 2**). In addition, the population of *Alphaproteobacteria* was significantly decreased, while *Gammaproteobacteria* was significantly increased in all ciprofloxacin exposure groups as compared to the control group. Moreover, *Saprospirae* was significantly decreased only with ciprofloxacin exposure at the lower level of ciprofloxacin residue in soil treatment group (0.04 mg/kg) as compared to the control group (Supplementary Table S2).

Based on sequence identification, a further evaluation was performed focusing on bacterial that might be associated with pathogeneses and ciprofloxacin resistance. For example, *Agrobacterium*, which belongs to *Alphaproteobacteria*, and *Clostridium*, which belongs to *Clostridia*, were significantly decreased in the 4 and 0.4 mg/kg ciprofloxacin concentrations treatment as compared to the control group. In addition, the abundance of *Xanthomonadales, which* belongs to *Gammaproteobacteria*, *Bacillales, which* belongs to *Bacillales*, and *Burkholderia*, which belongs to *Betaproteobacteria*, were greater in the 0.04 mg/kg ciprofloxacin exposure group than the 0.4 and 4 mg/kg ciprofloxacin groups (Supplementary Table S2). Raw sequence were submitted to Sequence Read Archive database in NCBI (accession No. SAMN05575008, SAMN05575009, SAMN05575010, SAMN05575011).

## Discussion

The concentration of antibiotic residues was observed to be significantly correlated with variations in the bacterial community. For example, a positive correlation was observed between the concentration of sulfamethoxazole and tetracycline and the classes *Bacteroidia* and *Gammaproteobacteria* (Varela et al., 2014). In our study, we found that the abundance of the bacteria in the lower level of ciprofloxacin residue in soil treatment group is significantly distinct from the standard dose of ciprofloxacin for treatment. For instance, *Actinobacteria*, *Bacilli*, *Clostridia* and *Gammaproteobacteria* enriched, while *Acidobacteria* and *Holophagae* were inhibited in the lower level of ciprofloxacin residue in soil treatment group vs. the standard dose of ciprofloxacin for treatment. In addition, the abundance of *Xanthomonadales*, *Bacillales, Burkholderia*, and *Agrobacterium* were greater in the lower level of ciprofloxacin residue in soil treatment group than other level of ciprofloxacin groups. Previous study reported that *Xanthomonadales*, *Burkholderia cepacia* and *Bacillales* were associated with intrinsic ciprofloxacin resistance (Fajardo et al., 2008; Walsh and Duffy, 2013). *Agrobacterium* can be responsible for opportunistic infections in humans with weakened immune systems (Dunne et al., 1993; Hulse et al., 1993). These results perhaps suggested that the low ciprofloxacin concentration in soil is benefit for emergence and enrichment of ciprofloxacin-resistant bacteria. On the other hand, our studies also demonstrated that opportunistic pathogens such as *Enterococcus* increased in the lower level of ciprofloxacin residue in soil treatment group as compared with other groups. These data suggest that lower concentrations of ciprofloxacin in soil may increase bacterial pathogenesis and cause a threat to both human and animal health.

Recent studies from other laboratories showed that the development of resistant bacteria can occur at sub-MIC levels of antibiotic exposure from sensitive bacterial strains (Gullberg et al., 2011, 2014; Vega and Gore, 2014). According to Clinical and Laboratory Standards Institute guidelines (CLSI, 2015), the MIC of sensitive *Pseudomonas aeruginosa*, *Acinetobacter* spp., *Staphylococcus* spp., *Enterococcus* spp., *Haemophilus influenzae*, and *Haemophilus parainfluenzae* are ≤1 μg/ml, the MIC of sensitive *Salmonella* spp. and *Neisseria gonorrhoeae* are ≤0.06 μg/ml, the MIC of sensitive *Neisseria meningitidi*s are ≤0.03 μg/ml, and the MIC of sensitive *Yersinia pestis* are ≤0.25 μg/ml. In our current studies, there were detectable increased in the proportion of bacteria cultured from 4 mg/L ciprofloxacin medium in the lower level of ciprofloxacin residue in soil treatment group as compared to the standard dose of ciprofloxacin for treatment. These observations are consistent with other report indicating that low antibiotic levels, which are present in many natural environments, are relevant for the enrichment and maintenance of pre-existing antimicrobial resistant bacteria (Batt et al., 2006; Gullberg et al., 2011).

Additionally, previous studies have suggested that manure bacteria might not be well-adapted to the soil environment and therefore may decrease in abundance after application (Heuer et al., 2008). This outcome may depend on varying environmental conditions, such as temperature, oxygen, moisture, pH and the indigenous community present (Sengeløv et al., 2003; Heuer et al., 2008; Chee-Sanford et al., 2009). For example, the numbers of tetracycline-resistant bacteria increased in soil at the time when the manure was added. However, these increased antibiotic-resistant bacteria declined after the cessation of slurry applications to a level of non-slurry fertilized soil within 8 months (Sengeløv et al., 2003). In the current studies, we showed that the relative abundance of PMQR genes significantly dissipated at day 60 which is consistent with the above investigations in other laboratories (Sengeløv et al., 2003; Heuer et al., 2008; Chee-Sanford et al., 2009). In addition, it has been reported that ciprofloxacin at approximately fivefold higher concentrations than its minimum detectable level in biosolid compost feedstock had no effect on the enrichment of CIPr genes (Youngquist et al., 2014). Our studies also showed that the abundance of PMQR genes dissipated faster in the standard dose of ciprofloxacin for treatment (dissipation rate 120.2–142.8%) vs. the low ciprofloxacin (dissipation rate 88.5–106.6%), suggesting that high ciprofiloxacin concentration had less influence on the enrichment of PMQR genes.

## Conclusion

Lower ciprofloxacin concentration incorporated into the soil treated with manure influenced not only the dissipation of PMQR genes, especially *qnrS*, *oqxA* and *aac (6^′^)-Ib-cr*, but also changed the bacterial taxa in the soil. The dissipation of PMQR genes in the presence of lower ciprofloxacin concentration was slower than with higher levels. Lower ciprofloxacin levels in the soil can provide a selective advantage for bacteria in orders including *Xanthomonadales* and *Bacillales* and genera including *Agrobacterium*, *Bacillus*, *Enterococcus*, and *Burkholderia*.

## Author Contributions

Y-HL, JS conceived of this study and participated in its design and coordination. TH designed the experiment and drafted the manuscript. TH, YX, and JZ carried out the detection on the relative quantification of ARGs and enumeration of ciprofloxacin-resistant bacteria. X-PL carried out the amplicon sequencing. D-HZ carried out the UPLC–MS/MS determination. LL participated in the statistical analyses. All authors read and approved the final manuscript.

## Conflict of Interest Statement

The authors declare that the research was conducted in the absence of any commercial or financial relationships that could be construed as a potential conflict of interest.

## Supplementary Material

FIGURE S1 **Cultivation-based estimation of the relative abundance of ciprofloxacin-resistant bacteria in all groups isolated from days 0, 30, and 60.** ^a,b^Significantly different (black for group A, red for group B, blue for group C, and green for group D).
