# Supplementary figures and images for: Low-Concentration Ciprofloxacin Selects Plasmid-Mediated Quinolone Resistance Encoding Genes and Affects Bacterial Taxa in Soil Containing Manure
Source: Front Microbiol. 2016 Nov 1;7:1730. doi: 10.3389/fmicb.2016.01730 (PMC5088497; doi:10.3389/fmicb.2016.01730)

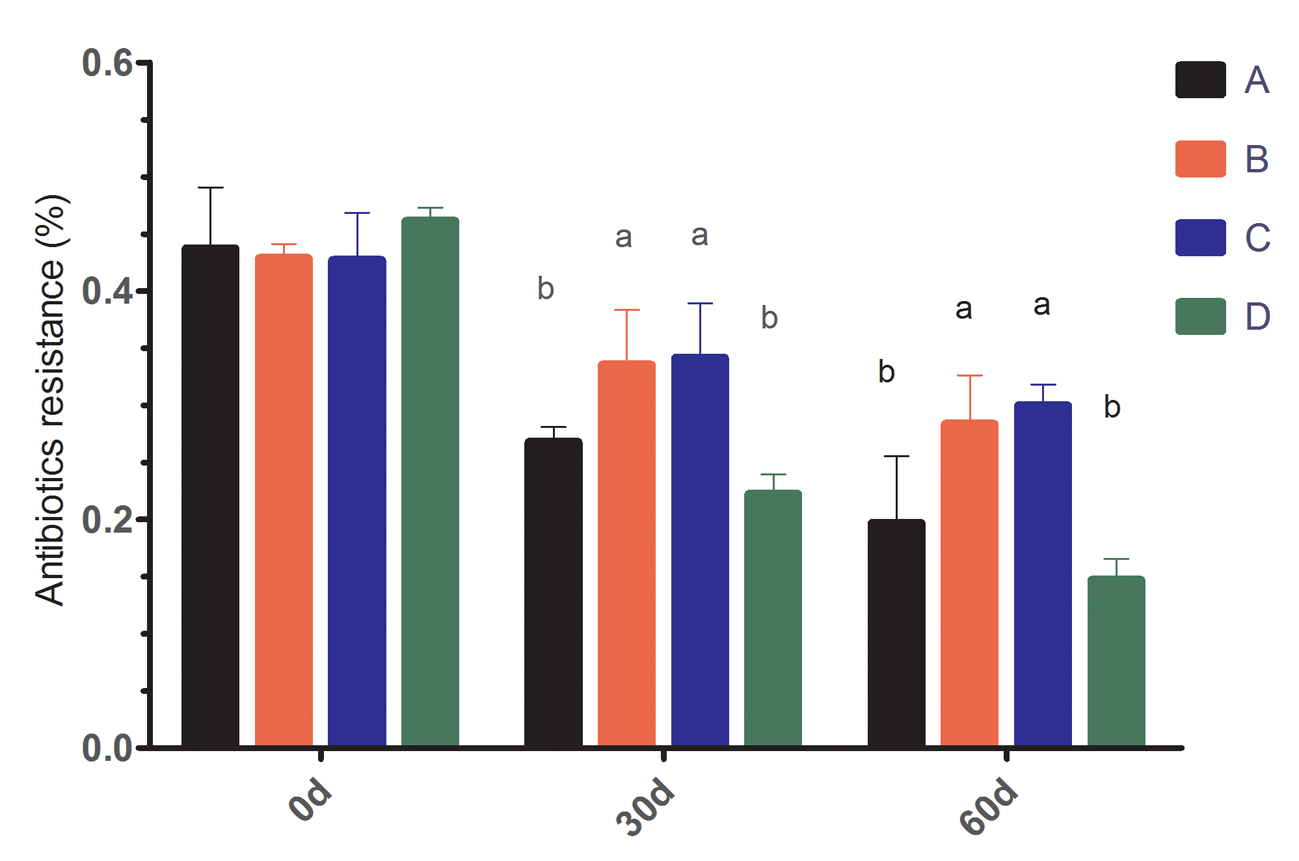

Supplement: FIGURE S1 — Cultivation-based estimation of the relative abundance of ciprofloxacin-resistant bacteria in all groups isolated from days 0, 30, and 60. a,bSignificantly different (black for group A, red for group B, blue for group C, and green for group D). [file Image_1.TIF]
