# Supplementary material for: Low-Concentration Ciprofloxacin Selects Plasmid-Mediated Quinolone Resistance Encoding Genes and Affects Bacterial Taxa in Soil Containing Manure
Source: Front Microbiol. 2016 Nov 1;7:1730. doi: 10.3389/fmicb.2016.01730 (PMC5088497; doi:10.3389/fmicb.2016.01730)
Supplement: Supplementary file 2 [file Table_1.DOCX]

Table S1 Relative abundance of PMQR genes in treatment and control groups.

| days | treatments | PMQR genes | | |
| --- | --- | --- | --- | --- |
|  |  | *qnrS* | *oqxA* | *aac(6’)-Ib-cr* |
| 0 | Group A | 3.20E-02±1.21E-03^a^ | 7.34E-01±2.83E-02^a^ | 3.07E-01±2.47E-02^a^ |
|  | Group B | 3.33E-02±6.08E-04^a^ | 7.57E-01±9.17E-03^a^ | 3.23E-01±2.44E-02^a^ |
|  | Group C | 2.94E-02±1.19E-03^b^ | 7.19E-01±1.69E-02^a^ | 3.00E-01±3.59E-02^a^ |
|  | Group D | 3.22E-02±1.25E-03^a^ | 8.58E-01±1.63E-02^a^ | 2.85E-01±1.20E-02^a^ |
| 30 | Group A | 9.44E-04±2.67E-05^d^ | 2.02E-02±1.53E-04^d^ | 9.54E-03±4.60E-04^d^ |
|  | Group B | 8.33E-03±1.53E-05^a^ | 7.91E-02±6.81E-04^a^ | 2.66E-02±6.51E-04^b^ |
|  | Group C | 6.33E-03±1.53E-05^b^ | 7.45E-02±2.90E-03^b^ | 2.49E-02±4.58E-04^c^ |
|  | Group D | 4.24E-03±2.65E-05^c^ | 5.45E-02±3.60E-03^c^ | 3.25E-02±3.22E-03^a^ |
| 60 | Group A | 4.47E-05±2.60E-06^d^ | 2.42E-03±4.05E-04^d^ | 1.21E-03±1.61E-04^d^ |
|  | Group B | 4.16E-04±3.79E-06^a^ | 3.14E-02±3.21E-04^a^ | 6.71E-03±9.06E-04^a^ |
|  | Group C | 2.16E-04±3.79E-06^b^ | 1.88E-02±4.93E-04^b^ | 5.09E-03±3.72E-04^b^ |
|  | Group D | 8.47E-05±2.60E-06^c^ | 6.48E-03±2.08E-04^c^ | 2.97E-03±5.57E-05^c^ |

PMQR genes: plasmid-mediated quinolone resistance gens. *qnrA*, *qnrB*, *qnrC*, *qnrD* and *qepA* were absent. Different letters mean significant difference (*p* < 0.05) between different groups in a certain time point.
