# Supplementary material for: Low-Concentration Ciprofloxacin Selects Plasmid-Mediated Quinolone Resistance Encoding Genes and Affects Bacterial Taxa in Soil Containing Manure
Source: Front Microbiol. 2016 Nov 1;7:1730. doi: 10.3389/fmicb.2016.01730 (PMC5088497; doi:10.3389/fmicb.2016.01730)
Supplement: Supplementary file 3 [file Table_2.DOCX]

Table S2 Variation in the relative abundance of bacterial classes, orders associated with ciprofloxacin-resistant bacteria and genera associated with opportunistic pathogens in treated samples compared to control samples.

| Bacteria taxa | Sample and abundance (%) | | | |  |
| --- | --- | --- | --- | --- | --- |
| Classes | A | B | C | D | |
| *Acidobacteriia* | 6.12^a^ | 5.95^a^ | 5.90^a^ | 3.31^b^ | |
| *Actinobacteria* | 0.45^a^ | 0.58^b^ | 0.86^c^ | 1.17^d^ | |
| *Alphaproteobacteria* | 35.08^a^ | 35.77^a^ | 35.08^a^ | 38.11^b^ | |
| *Bacilli* | 6.04^a^ | 5.04^b^ | 7.42^c^ | 6.23^a^ | |
| *Betaproteobacteria* | 4.50^a^ | 3.97^b^ | 4.75^a^ | 3.43^c^ | |
| *Clostridia* | 2.49^a^ | 2.38^a^ | 4.56^b^ | 5.39^c^ | |
| *Deltaproteobacteria* | 2.00^a^ | 1.95^a^ | 2.33^b^ | 2.17^a,b^ | |
| *Gammaproteobacteria* | 15.46^a^ | 14.15^b^ | 17.21^c^ | 13.49^d^ | |
| *Holophagae* | 0.66^a^ | 0.53^a,b^ | 0.49^b^ | 0.24^c^ | |
| *Pedosphaerae* | 0.72^a^ | 1.13^b^ | 0.60^a^ | 0.72^a^ | |
| *Phycisphaerae* | 0.76^a^ | 0.76^a^ | 0.65^a^ | 0.51^b^ | |
| *Planctomycetia* | 0.92^a^ | 1.29^b^ | 0.98^a,c^ | 1.09^c^ | |
| *Saprospirae* | 13.58^a^ | 14.08^a,b^ | 11.01^c^ | 14.28^b^ | |
| *Solibacteres* | 4.05^a^ | 3.74^a,b^ | 3.11^c^ | 3.58^b^ | |
| *Spartobacteria* | 1.35^a.b^ | 1.86^c^ | 1.26^b^ | 1.55^a^ | |
| *Sphingobacteriia* | 1.47^a^ | 2.36^b^ | 0.78^c^ | 1.27^d^ | |
| Order |  |  |  |  | |
| *Xanthomonadales* | 14.44^a^ | 12.87^b^ | 15.33^c^ | 11.58^d^ | |
| *Bacillales* | 1.38^a^ | 1.27^a^ | 1.71^b^ | 1.44^a^ | |
| Genus |  |  |  |  | |
| *Acinetobacter* | 0.12^a^ | 0.14^a^ | 0.15^a^ | 0.16^a^ | |
| *Agrobacterium* | 0.21^a^ | 0.18^a^ | 0.62^b^ | 0.44^c^ | |
| *Bacillus* | 0.96^a,b^ | 0.92^a,b^ | 1.06^b^ | 0.81^a^ | |
| *Clostridium* | 0.31^a^ | 0.23^a^ | 0.45^b^ | 0.57^b^ | |
| *Enterococcus* | 4.28^a^ | 3.51^b^ | 5.11^c^ | 4.31^a^ | |
| *Escherichia* | 0.11^a,b^ | 0.12^a,b^ | 0.08^b^ | 0.15^a^ | |
| *Sphingomonas* | 1.02^a^ | 1.14^a^ | 0.98^a^ | 1.02^a^ | |
| *Burkholderia* | 0.39^a^ | 0.27^b^ | 0.43^a^ | 0.23^b^ | |

Different letters ^a,b,c,d^ mean significant difference (p< 0.05) between different groups.
